# Participation Education Intervention for People With SCI From LMIC: A Pilot Randomized Clinical Trial

**DOI:** 10.1155/oti/8774897

**Published:** 2026-05-21

**Authors:** Moussa Kleib Abumostafa, Nicola Ann Plastow, Maggi Savin-Baden

**Affiliations:** ^1^ Occupational Therapy Department, Hamad Rehabilitation Hospital, Gaza, State of Palestine; ^2^ Division of Occupational Therapy, Stellenbosch University, Cape Town, South Africa, sun.ac.za; ^3^ Blackfriars Hall, University of Oxford, Oxford, UK, ox.ac.uk

**Keywords:** clinical trials, daily occupations, participation, SADL-eM, spinal cord injury

## Abstract

**Introduction:**

Spinal cord injury (SCI) is associated with inequality‐related participation restrictions. Participation education has been proposed as an intervention to promote participation in daily occupations. This pilot randomized clinical trial (RCT) evaluates the feasibility and minimal effect of the SADL‐eM, a participation educational intervention, on the participation of people with spinal cord injury (PW‐SCI) in daily occupations.

**Methods:**

A two‐arm pilot RCT included 32 PW‐SCI from three inpatient rehabilitation settings in the Gaza Strip aged 18–65 years. Eligible cases were randomized equally into two groups. Both groups received standard care. The intervention group also used the SADL‐eM during occupational therapy sessions. Participation and adherence were assessed at baseline and after 6 weeks. The Arabic version of the Spinal Cord Independence Measure‐Self Reported (SCIM‐SR‐Ar) was the primary outcome measure.

**Results:**

Demographic and injury characteristics were homogenous at baseline (*p* > 0.05). There was no difference between groups at baseline (*p* < 0.05). Comparing intervention to standard care, the SADL‐eM was insignificant after 6 weeks of intervention (*p* > 0.05) while the inpatient rehabilitation improved participation in daily occupations (16.06%–17.00%) in the total sample (*p* ≤ 0.001).

**Conclusion:**

There was an improvement in all outcome measures used; however, the SADL‐eM did not reach significance. The intended RCT has major barriers, for example, a low recruitment rate. Community settings can be unique for such an RCT.

**Trial Registration:**

ClinicalTrials.gov identifier: NCT04735887

## 1. Introduction

Trauma, falling, road traffic incidents, gunshot, and stab wounds are established as the main cause of spinal cord injury (SCI) (68.8%) in low‐ and middle‐income countries (LMICs) ([1, 2]; Nisari Ziba et al.[3]; [4]). Participation education focused on activities of daily living (ADLs) has been shown to reduce participation restrictions among people with spinal cord injury (PW‐SCI) [5]. However, there is limited evidence on its application and effectiveness among PW‐SCI in LMICs and especially those affected by armed conflict and war, such as PW‐SCI living in the Gaza Strip of Palestine [6, 7]. This evidence gap is significant, as this population faces a high risk of participation inequality. Kader et al. [8], Reinhardt et al. [9], and Yao et al. [10] highlighted specific barriers to rehabilitation in LMICs, which increase the risk of participation inequality. These barriers include a short length of hospital stay; limited access to inpatient rehabilitation; long waiting lists; limited community‐based rehabilitation and assistive devices; limited access to the internet and new technology; environmental barriers restricting ambulation; residing in a rural area; unemployment; and limited skills to deal with spasticity, pain, incontinence, and sexual health and fertility needs. Participation inequality for PW‐SCI is further exacerbated by limited opportunities for employment, unfair salaries and reward systems for people with disabilities, stigma, poor economic circumstances, and alienation [6, 7].

Participation has emerged as an important outcome of the rehabilitation of PW‐SCI [11]. The International Classification of Functioning, Disability and Health (ICF), developed by the World Health Organization (WHO), defines participation as involvement in a life situation or occupations such as interpersonal relationships, employment, education, and community life occupations [12]. Participation represents the societal perspective of functioning and emphasizes the person′s interaction with their environment and their performance within a social context to fulfill individual and collective roles. It can be restricted when an individual experiences problems or barriers in performing these social roles compared to an individual without a similar problem or health condition [12]. Since activity limitation causes participation restriction, the ICF combined activities and participation into a single taxonomy and described nine scales of participation and activities. These are as follows: learning and applying knowledge; general tasks of living; interpersonal communication; mobility; self‐care; domestic life; interpersonal relationships; major life areas (education, work, and economic); and community, civic, and social life [13]. While participation shares aspects of ADL, the ICF maintained a clear conceptual and operational distinction between activity and participation [14–16].

Reducing participation inequality for PW‐SCI living in LMICs like the Gaza Strip, Palestine, is important because participation is related to health equity [17] and occupational justice issues [18, 19]. Activity limitations are difficulties in executing ADL, while participation restrictions are problems experienced in life situations because of activity limitations. Thus, activity limitations are viewed as an indicator of participation inequality. PW‐SCI are more likely to be dependent on caregivers for self‐care activities such as toileting and preventing secondary complications like pressure injuries. However, participation inequality extends to other occupations beyond basic ADLs. Gross‐Hemmi et al. [2] found that participation in productivity, leisure, and social activities dropped to 34.5% of preinjury levels following SCI. The most significant restrictions were observed in employment, education, sports, and partner relationships.

While Javanmard et al. [15] review identified six outcome measures for participation of PW‐SCI, Noonan et al. [16] review identified eight outcome measures for participation of PW‐SCI; the tools assess aspects of mobility, ADL, recreation/leisure, and work/education. Among these tools, the Incontinence–Activity Participation Scale, WHODASII, and Reintegration to Normal Living Index (RNL) instruments included aspects of ADL as a specific domain. Despite this, Javanmard et al. and Noonan et al. maintained a clear conceptual distinction between activity and participation: Activity is defined as the routine actions essential for survival and well‐being, while participation refers to engagement in broader life situations. These authors argued that activity limitations in SCI directly result in participation restrictions and societal inequality. The Spinal Cord Independence Measure‐III (SCIM‐III) includes mobility and self‐care domains [20], which are aspects of participation; however, Javanmard et al. and Noonan et al. did not consider the SCIM‐III, a participation outcome measure based on its conceptual foundation. However, the SCIM‐III, which primarily measures activity limitation, was utilized in this study as a proxy indicator and a primary outcome measure for assessing participation restriction and inequality among persons with SCI, as a unique outcome ability measure of PW‐SCI focusing on what a person can actually do.

Research about SCI is aimed at optimizing treatment outcomes, for example, motor recovery, functionality, participation in daily occupations, quality of life, and inclusion [1, 21]. Further work is needed to reduce participation inequality for PW‐SCI in LMICs and areas affected by armed conflict and war. A systematic review by [22]) emphasized the importance of working in partnership with PW‐SCI when developing specific tools or interventions and conducting relevant research. Partnership promotes participation in daily occupations and alleviates inequality, thereby providing a means to also promote occupational justice [23]. A partnership approach may include coexploring the gaps in service delivery; identifying needs; and planning, implementing, and disseminating solutions. Participation education is one intervention for PW‐SCI that uses a wide range of constructed learning means, including education manuals, online media, expert peers, videos, videoconferencing, handouts, lectures, discussion sessions, leaflets, mobile applications, and presentations (Rodger and Bench [24]). Participation education is aimed at optimizing rehabilitation outcomes such as participation in daily occupations and equality [6, 7] and improving the quality of life and satisfaction for PW‐SCI [4].

A recent example of a partnership approach to intervention development that promoted occupational justice was the codevelopment of the spinal cord injury activities of daily living Education Manual for (SADL‐eM) PW‐SCI using participatory action research [6, 7]. In that study, 54 experts in SCI rehabilitation from the Gaza Strip of Palestine codeveloped a participation education intervention. However, few studies have evaluated the effect of participation education on participation, especially in LMICs ([6, 7]. We, therefore, developed a protocol for an RCT to evaluate the effectiveness of the SADL‐eM on participation for adult inpatients receiving rehabilitation at one of three hospitals in Gaza, Palestine [6, 7]. The researchers then conducted a feasibility study that evaluated the usability of the SADL‐eM intervention for this population, participation in a planned RCT, and the properties of selected outcome measures, with PW‐SCI who had already completed their rehabilitation [5]. That feasibility study found that the SADL‐eM was an implementable participation education tool, with sufficient demand and high interest from PW‐SCI. The next step was to evaluate the feasibility of a full‐scale RCT of the intervention with current rehabilitation inpatients.

A pilot RCT is an acknowledged method of research to test intervention feasibility [25]. Many pilot RCTs have been conducted to test a variety of interventions for PW‐SCI, for example, a mobile application to improve healthy lifestyles [26], massage pain management [27, 28], and internet‐delivered mindfulness training [29]. However, the researchers were unable to identify any previous pilot RCT of educational interventions for PW‐SCI that aimed to improve participation in daily occupations. Also, most of the previous pilot RCTs were conducted in HICS, while only one was conducted in Bangladesh as an LMIC and included 30 people with new SCI [30].

### 1.1. Purpose

Our pilot RCT evaluated the likelihood of conducting a large‐scale RCT to evaluate the effect of an educational intervention on participation in three clinical rehabilitation settings in the Gaza Strip, Palestine. It identified possible barriers and suggested strategies to improve the conduct of the main RCT. This pilot trial also tested the minimal effect size of the SADL‐eM on participation to decide if it is worthwhile conducting the main RCT. Any changes in our primary outcome measures between the two study groups would be an important marker to proceed to the large‐scale RCT.

### 1.2. Research Question

This study adopted two questions: (a) “Was it possible to conduct a pilot RCT to evaluate the effect of the SADL‐eM on participation in clinical rehabilitation settings within LMIC?” and (b) “What was the minimal effect of the SADL‐eM on participation?”

### 1.3. Objectives

The researchers aimed to evaluate the pilot RCT feasibility: recruitment, retention of participants, implementation of eligibility criteria, the randomization process, participation education determinants (providers, time, frequency, content, was the SADL‐eM enough, and any changes), adherence to participation education intervention, and suitability of statistical tests. They also evaluated the preliminary effect of the SADL‐eM on the participation of PW‐SCI from LMIC. The researchers suggested strategies to improve the components of the main RCT.

### 1.4. Research Hypothesis

The study adopted two hypotheses. The null hypothesis assumes there is no participation outcome difference between the SADL‐eM and the standard care delivered to inpatients with SCI in the inpatient rehabilitation settings. The alternative hypothesis assumes that the implementation of a SADL‐eM delivered to inpatients with SCI in an inpatient rehabilitation setting improves their participation more than the standard care of rehabilitation.

## 2. Methods

### 2.1. Study Design

A pilot clinical trial is a preliminary trial that evaluates the feasibility of an intervention, sampling procedure, eligibility, randomization, participant retention, outcome measures, data collection process, and statistical analysis. The results are used to improve the trial components before a larger scale study is conducted. A pilot clinical trial may also test the initial effect of an intervention on a small sample to decide if the main trial is worthwhile, as in our study [25].

This is a pilot RCT (IRRID: [6, 7]) of two parallel arms, intervention and control, with a pre‐/posttest to evaluate a hypothesis of a cause‐and‐effect relationship. Participation education intervention was an independent variable, while participation in daily occupations was a dependent variable. This study used the Consolidated Standards of Reporting Trials (CONSORT) statement as the proposed standard for the reporting of parallel‐group RCTs [31].

### 2.2. Participants and Recruitment

The study adopted strict inclusion criteria to improve participants′ matching, reduce bias, and improve the findings′ reliability. Participants in the study were inpatients with SCI from the Gaza Strip, of both genders, of any cause or type of SCI. They were recruited from inpatients admitted to one of the three inpatient rehabilitation facilities in the Gaza Strip: Hamad, ElWafa, or ElAmal rehabilitation hospitals. Participants were individuals admitted for rehabilitation after a newly acquired SCI that occurred within the past 6 months. Readmissions were only eligible if their injury was sustained within the past 6 months. On the day of admission of a patient with SCI to inpatient rehabilitation, a research coordinator recruited cases, applied study eligibility criteria, and enrolled participants in one group of the pilot RCT. Participants were strictly assigned to study groups based on a random list that was generated online before the study started. The inclusion and exclusion criteria are listed in Table 1.

**Table 1:** Eligibility criteria.

Inclusion criteria	Exclusion criteria
Confirmed diagnosis of SCI by computerized tomography or magnetic resonance imaging techniques.	A concurrent injury, such as traumatic brain injury or other comorbidities restricting rehabilitation, such as deep vein thrombosis.
Admission to an inpatient rehabilitation setting.	History of addiction and substance abuse or psychiatric drug use in the past 6 months.
Willingness to participate in the pilot RCT.	Inactive involvement in a rehabilitation program.
Currently living in the Gaza Strip.	Persons with complete tetraplegia C4 or above.
Good Arabic literacy.	SCI lasted more than 6 months.
Age of 18–65 years.	Age below 18 or above 65 years.
ASIA: A, B, and C.	ASIA: D and E or persons who become walking ambulatory during the inpatient stage.
Stable medical condition.	Unstable medical condition.
Ability to be involved in an active rehabilitation course.	Persons on a mechanical ventilator.
The time elapsed after SCI was not more than 6 months.	The time of stay in inpatient rehabilitation is less than 6 weeks.
The minimum time of stay in inpatient rehabilitation is 6 weeks.	Communication and/or cognitive disorders.

### 2.3. Sample Size

The minimum sample size of the main RCT was 90 participants. This sample size calculation was based on 80% power, SD = 20*%*, effect size 15%, a confidence interval (CI) of 95%, and alpha = 0.05 (intervention group = 45 and control group = 45) [6, 7]. During the pilot RCT period (April 2021–May 2022), only 46 PW‐SCI were admitted for rehabilitation, and 32 were eligible for recruitment. Of these, 32 participants were enrolled in the study. This sample size was sufficient for the recommended 12–15 participants per treatment arm required to detect a medium effect in pilot trials, even when using an outcome variable featuring discrete categories with unequal intervals [32].

### 2.4. Study Setting

Government‐funded rehabilitation services for PW‐SCI were severely limited at the time of this research. The rehabilitation directorate in the Palestinian Ministry of Health (PMOH) recommends a short stay in inpatient rehabilitation to manage the long waiting list and the limited budget allocated for inpatient rehabilitation. This means that most PW‐SCI were prematurely discharged after becoming medically stable and before completing an inpatient rehabilitation program. Subsequently, PW‐SCI may receive low‐cost healthcare services provided by local charities, but these do not include inpatient rehabilitation. Health insurance is recommended in the Gaza Strip, but not compulsory. PW‐SCI who require rehabilitation services and do not have health insurance can pay directly for the rehabilitation services at private facilities.

There were only three clinical settings that provided postacute inpatient rehabilitation in the Gaza Strip of Palestine during our pilot RCT. All three were used to recruit participants for this study: Hamad, ElWafa, and ElAmal rehabilitation hospitals. These three inpatient rehabilitation settings were private healthcare facilities and provided postacute comprehensive rehabilitation services to people from the Gaza Strip. The inpatient rehabilitation focused on gaining physical functioning and addressing the minimum participation needs for returning to the community. Rehabilitation was based on individual care plans and a short 6‐week period of stay after injury. This could be a barrier to meaningful change in participation in daily occupations. However, what was unique in these rehabilitation settings was that inpatient services were provided 5 days a week (Sunday–Thursday), while on Friday and Saturday, patients went home. This offered rich opportunities to practice their daily occupations at home, as instructed by their therapists. Patients returned on Sunday mornings with rich feedback about their experiences of participation in daily activities at home. This feedback guided the rehabilitation team to plan for the next week. The rehabilitation teams in these settings included physical rehabilitation doctors, rehabilitation nurses, physiotherapists, occupational therapists, speech therapists, clinical dieticians, psychologists, social workers, orthotists, and community‐based rehabilitation workers.

### 2.5. SADL‐eM

The study used the SADL‐eM Arabic participation education intervention that includes 91 pages divided into six sections: (1) introduction, (2) rehabilitation team and its roles, (3) independence in ADL in the Gaza context after restrictions caused by SCI, (4) essential assistive devices for PW‐SCI, (5) potential home environment adaptations, and (6) guide to community resources. The text was supported with illustrative and contextually relevant pictures. Good Arabic literacy is required for the use of the SADL‐eM. The SADL‐eM is intended in this study to improve participation in daily occupations; however, other health aspects can be improved and positively affect participation, for example, prevention of pressure injuries and UTI, and improving sexual health [6, 7].

The principal researcher conducted training for six occupational therapists (three males and three females) over 4 h about how to administer the SADL‐eM during occupational therapy sessions [6, 7]. These occupational therapists were selected based on their convenience. They included the SADL‐eM in the treatment of intervention group participants for 6 weeks. Each participant received three educational sessions or more per week, with 15 min for each session as a minimum. To offer choice to participation, participants selected the part of the manual to be discussed in each session in collaboration with their treating occupational therapist. However, all aspects of the manual were addressed, ensuring that all participants received all of the information. Each participant received an individual treatment to reduce contamination bias. The use of the SADL‐eM did not include additional requirements. The participants in the intervention group kept the education manual to review between occupational therapy sessions and after discharge from the rehabilitation setting as a reference.

### 2.6. Outcome Measures

Outcome measures included the Arabic version of the Spinal Cord Independence Measure‐Self Reported (SCIM‐SR‐Ar) as the primary outcome measure and the third version of the SCIM‐III, Private Religiousness Practice subscale (PRPS), Organizational Religiousness Practices subscales (ORSF), questions about other ADL domains covered by the education manual, and a patient adherence questionnaire (PAQ) as secondary measures.

The SCIM‐SR‐Ar and SCIM‐III are 17‐item tools that evaluate the ability of PW‐SCI to participate in self‐care, sphincter and respiration, and mobility activities independently, with assistance, or with assistive devices. These three domains represent basic ADLs that are needed to support participation in other occupations. The SCIM‐III is therapist‐administered [20], while the SCIM‐SR‐Ar is a patient‐rated outcome measure (PROM). A recent scoping review of patient‐reported outcome measures of functioning, disability, and health for PW‐SCI confirmed that the SCIM‐SR is among the most frequently applied PROMs, and its psychometric properties have been evaluated the most thoroughly [33].

The inpatient rehabilitation course included 5 days of inpatient rehabilitation and 2 days of community integration a week. This unique occupational context and occupational participation of our participants meant that we needed to consider participation beyond the three domains measured by these tools. In addition to self‐care and mobility, Abumostafa et al. [6, 7] also identified leisure, education, productive work, religious activities, and intimate relationships as important domains of participation for PW‐SCI living in the Gaza Strip, Palestine.

The Brief Multidimensional Measure of Religiousness/Spirituality (BMMRS) is a 38‐item self‐report measure of religiousness or spirituality across 11 domains. The researchers were interested in private religious practices and attending organizational religiousness activities to measure the frequency of an individual′s involvement in religious activities. The original version of the PRPS included four questions [34, 35]. The original version of the ORSF included two questions to measure the involvement of the individual in a formal public religious institution such as a church or mosque [34, 35]. The researchers added another question for each subscale to improve reliability based on the findings of a previous feasibility study and included the mosque as a place of worship and the Quran as a religious text [5]. Neither of the subscales was available in Arabic.

Driving and community mobility and health management were two other important participation domains. We, therefore, developed two questions to capture participants′ perceptions of their performance in each domain. This short questionnaire was used in a previous feasibility study and showed very high reliability (Cronbach′s alpha: 0.90–0.97) [5].

The PAQ is a therapist‐administered tool, and it was developed for the sake of this pilot RCT. The PAQ evaluated the adherence of the participants in the intervention group to the SADL‐eM. The tool included a table that captured data about time determinants (number, time, and frequency of educational sessions) and eight questions on participants′ commitment to the content (topics and order) of the intervention [5].

### 2.7. Data Collection

Data were collected using seven separate questionnaires. The principal researcher translated the English outcome tools (biographical data, SCIM‐III, private religious practices, attending organizational religiousness, and the other three domains of ADL), which were tested in the feasibility study [5]. The principal researcher trained three research assistants (physiotherapists) to administer these tools and collect data. Consenting and biographical data were collected once upon admission to the study and before allocation to study groups. SCIM‐SR‐Ar, SCIM‐III, religiousness subscales, and the three ADL domain questions were collected twice after allocation to study groups and 6 weeks later. The research assistant read the questions in English for all tools in English and used the Arabic translation to explain the questions during the interview, while the SCIM‐SR‐Ar was patient‐administered. Data collection continued until the desired number of cases was reached. The same outcome measures were used throughout the study.

The PAQ was a therapist‐reported tool completed by the treating occupational therapist in two stages. First, during 6 weeks of intervention, the educational sessions using the SADL‐eM were recorded in minutes immediately after each session. Second, after 6 weeks of intervention, the occupational therapist evaluated the adherence of participants to the use of the SADL‐eM over the past 6 weeks.

### 2.8. RCT Feasibility

To determine the feasibility of the planned RCT, the researchers were interested in recruitment (> 60%), the retention of participants (> 70%), the application of eligibility criteria, adherence to randomization methods, adherence to the treatment protocol of the SADL‐eM, and the suitability of statistical tests [25].

### 2.9. The Substantial Effect of the Intervention Used

Scivoletto et al. [36] pointed out that a 10% improvement in SCIM‐III due to standard care of rehabilitation is a clinically significant difference and worthwhile patient outcome. The researchers decided to accept 20% overall improvement in SCIM‐III or SCIM‐SR‐Ar due to SADL‐eM, besides standard care of rehabilitation, in the intervention group after 6 weeks of inpatient rehabilitation. The researchers built on a 10% additional difference in SCIM‐III or SCIM‐SR‐Ar between the intervention and control groups after 6 weeks of intervention.

### 2.10. Ethical Considerations

The study was approved by the Stellenbosch University Human Research Ethics Committee (HREC Project ID: 1635) and Helsinki Committee for Ethical Approval (PHRC/HC/689/20). The enrollment of participants in the study was voluntary, and the participants did not receive any financial incentive. All study participants were provided with the necessary information about the study purpose and process, and their questions were answered by the principal researcher and research assistants. Written consent was sought from each participant before any data collection. Freedom of participation, refusal, or withdrawal was assured at any stage of the pilot RCT. The study was risk‐free and did not bear any discomfort to participants whose anonymity and confidentiality were assured in the consent form and sustained throughout the study. Data collection documents were coded to prevent disclosure and maintain participants′ anonymity and confidentiality. The participants in the control group received a free copy of the SADL‐eM and an explanation after the study completion.

### 2.11. COVID‐19 Considerations

The research assistants and participants followed the required coronavirus pandemic (COVID‐19) precaution guidelines published by the WHO and applied by each facility [37]. They used medical disposable latex gloves, face shields, surgical masks, and hand hygiene alcohol gel.

### 2.12. Data Analysis

IBM‐SPSS (21) software was used to perform descriptive statistics and run the necessary parametric and nonparametric tests to answer the research questions and test study hypotheses. The researchers set the CI at 95% and alpha to 0.05 during all statistical tests. Homogeneity between the intervention and control groups was tested using chi‐square tests, Fisher′s exact test, and independent *t*‐tests. The normality of data distribution was evaluated using the Kolmogorov–Smirnov test. The between‐group mean differences at 6 weeks after the educational intervention were analyzed using linear regression analysis.

## 3. Results

### 3.1. Participants

**Figure 1:**
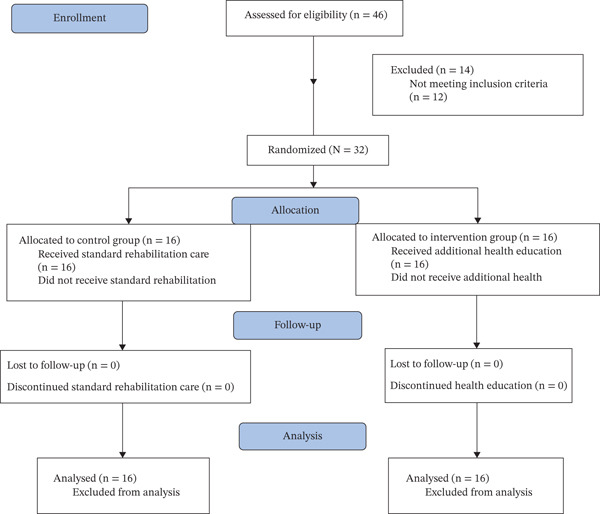
Study flow diagram (CONSORT statement).

Forty‐six PW‐SCI were admitted to inpatient rehabilitation in Gaza during the study timeframe. Thirty‐two (70%) were found to be eligible and agreed to participate in the pilot RCT (see Figure 1). Fourteen cases (30%) were ineligible: four sustained injury more than 6 months before recruitment, two had high quadriplegia and poor potential for rehabilitation, one had ASIA E and was able to walk, one had a mental illness, one was below 18 years old, one had a 4‐week admission, one had multiple fractures in four extremities, one had an unstable medical condition due to a spinal cord tumor, and two refused participation.

Most participants were men (78.1%). The age of participants ranged from 18 to 65 years (M ± SD: 36.13 ± 15.33 years). Then, 65.6% of the participants were married before SCI. More than half were from the northern zone of the Gaza Strip. Although just less than half of the participants (43.8%) completed high school, 37.5% of participants had a university degree, either a diploma (15.6%), a bachelor′s (12.5%), or a postgraduate degree (9.4%). Half of the participants were employed before their SCI, whereas more than one‐third (37.5%) were unemployed. Only one was a student. Nearly half of the participants (43.8%) had technical and craft or routine manual and service occupations. At admission, half of the participants (50%) had a monthly income below 2000 INS (Israeli new shekel), approximately $600. This monthly income sustains a family of six to eight people. All the participants were Arabic native speakers and Muslims. Four of the participants were bilingual in Arabic/English.

Although the researchers expected to recruit participants from all three hospitals based on prepandemic admission patterns, 30 participants were recruited from Hamad Medical Rehabilitation Hospital, while two received rehabilitation care at ElWafa Medical Rehabilitation Hospital. Analysis of between‐group differences found no statistically significant differences (*p* > 0.05) in demographic characteristics between the intervention and control groups (see Table 2).

**Table 2:** Demographic and injury characteristics of participants (*n* = 32) and correlations with SCIM‐SR‐Ar.

Variables	Intervention group (*n* = 16)	Control group (*n* = 16)	Total sample (*n* = 32) (%)	Between‐group differences at baseline	
				Test statistic	*p* value
Demographic characteristics					
Gender				Fisher^′^s (1) = 1.00	0.500
Men	13	12	25 (78.1%)		
Women	3	4	7 (21.9%)		
Age: Mean ± SD (years)	34.81 ± 17.54	37.44 ± 13.19	36.13 ± 15.33	Normal distribution, *T* − test (1) = 2.80	0.105
18–39 years	11	10	21 (65.6%)		
40–60 years	2	4	6 (18.8%)		
> 60–65 years	3	2	5 (15.6%)		
Marital status				*X* ^2^ (1) = 0.139	0.710
Never married	6	5	11 (34.4%)		
Married	10	11	21 (65.6%)		
Address				*X* ^2^ (2) = 0.659	0.719
Northern zone	9	8	17 (53.1%)		
Middle zone	3	2	5 (15.6%)		
Southern zone	4	6	10 (31.3%)		
Level of education				Kruskal − Wallis (1) = 0.208	0.648
Postgraduate education	2	1	3 (9.4%)		
Bachelor′s degree	3	1	4 (12.5%)		
Diploma	1	4	5 (15.6%)		
Completed high school	5	9	14 (43.8%)		
Completed primary school	5	1	6 (18.8%)		
Employment status				*X* ^2^ (3) = 2.971	0.396
Unemployed	6	6	12 (37.5%)		
Employee	8	8	16 (50.0%)		
Self‐employed with employees	0	1	1 (3.1%)		
Self‐employed without employees	2	0	2 (6.3%)		
Student	0	1	1 (3.1%)		
Type of work				Kruskal − Wallis (2) = 1.186	0.276
Modern professional occupations	2	0	2 (6.3%)		
Senior managers or administrators	0	1	1 (3.1%)		
Technical and craft occupations	1	3	4 (12.5%)		
Semiroutine manual and service occupations	1	3	4 (12.5%)		
Routine manual and service occupations	4	2	6 (18.8%)		
Traditional professional occupations	2	0	2 (6.3%)		
Accommodation				Fisher^′^s (1) = 1.000	0.500
Informal	0	1	1 (3.1%)		
Formal	16	15	31 (96.9%)		
Living arrangements				Fisher^′^s (2) = 1.000	0.500
Living alone	0	1	1 (3.1%)		
Living with family	16	15	31 (96.9%)		
Monthly income before the current SCI				Kruskal − Wallis (3) = 0.030	0.862
< 1000 INS	4	5	9 (28.1%)		
1001–2000 INS	3	4	7 (21.9%)		
2001–3000 INS	2	2	4 (12.5%)		
> 3000 INS	1	1	2 (6.3%)		
Language proficiency				Fisher^′^s (3) = 1.000	0.700
Arabic	14	14	28 (87.5%)		
Arabic and English	2	2	4 (12.5%)		
Religion					
Muslim	16	16	32 (100.0%)		
Rehabilitation setting				*X* ^2^ (4) = 2.133	0.144
Hamad	15	15			
ElWafa	1	1			
AlAmal	0	0			
Injury characteristics					
Cause of current SCI				*X* ^2^ = 3.515	*p* = 0.476
Trauma			22 (68.8%)		
Disc degeneration			4 (12.5%)		
Tumor			3 (9.4%)		
Transverse myelitis			2 (6.3%)		
Infection			1 (3.1%)		
Undergoing surgery				Fisher^′^s = 1.000	*p* = 0.500
Yes			23 (71.9%)		
No			9 (28.1%)		
Type of SCI				Mann − Whitney = 120.00	*p* = 0.551
Incomplete			29 (90.6%)		
Complete			3 (9.4%)		
Dysfunction after SCI				Kruskal − Wallis = 1.130	*p* = 0.288
Paraparesis			16 (50.0%)		
Paraplegia			8 (25.0%)		
Quadriparesis			7 (21.9%)		
Quadriplegia			1 (3.1%)		
ASIA classification				Kruskal − Wallis = 2.258	*p* = 0.133
ASIA A			7 (21.9%)		
ASIA B			3 (9.4%)		
ASIA C			22 (68.8%)		
Level of SCI				Kruskal − Wallis = 0.178	*p* = 0.673
Cervical			9 (28.1%)		
Thoracic			10 (31.3%)		
Lumbar			13 (40.6%)		
Cause of current discharge				*X* ^2^ = 2.333	*p* = 0.311
Completed inpatient rehabilitation program			18 (84.4%)		
Referred to another hospital			1 (3.1%)		
Inadequate healthcare coverage			4 (12.5%)		

*Note:* *p* > 0.05, insignificant.

Abbreviation: INS, Israeli new shekel.

Causes of current SCI varied across participants, including trauma (68.8%), disc degeneration (12.5%), tumor (9.4%), transverse myelitis (6.3%), and infection (3.1%). Most injuries (40.6%) were lumbar, 31.3% thoracic, and 28.1% cervical. Then, 90.6% of participants had incomplete SCI, 50.0% experienced paraparesis, 25.0% paraplegia, 21.9% quadriparesis, and 3.1% quadriplegia. Rating using the American Spinal Injury Association Impairment Scale showed that 21.9% of participants had total impairment (ASIA A), 9.4% retained some sensation but no motor function below the lesion (ASIA B), and 68.8% of the participants had incomplete impairment below the lesion but were not strong enough to move against gravity (ASIA C). Most participants (71.9%) underwent surgery after the current SCI. The time between SCI onset and admission to an inpatient rehabilitation facility ranged from 1 to 24 weeks (M ± SD: 8.34 ± 7.61). The time between SCI onset and admission to rehabilitation was as long as 20–24 weeks in five cases because of injury severity and the medical instability of these participants. Only four participants had previous admissions to inpatient rehabilitation for a short length of stay of 1–4 weeks. Three participants received previous participation education from an occupational therapist that ranged from 14 to 25 sessions (M ± SD: 18.00 ± 6.08). The duration of participation education is unknown. Then, 84.4% of participants were discharged after they completed their inpatient rehabilitation program, while one (3.1%) was referred to another hospital for further management. Four (12.5%) had inadequate healthcare coverage and were discharged before completing their inpatient rehabilitation program. Analysis of between‐group differences found no statistically significant differences (*p* > 0.05) in injury characteristics between the intervention and control groups (see Table 2).

### 3.2. Randomized Clinical Trial Feasibility

The researchers were able to recruit 32 of 46 PW‐SCI admitted during the study period (70%). Only two refused to participate in the study, while the application of the eligibility criteria meant that 12 people were ineligible. The 32 participants were randomized equally in two study arms using simple randomization with a random list of numbers generated online before the recruitment of participants. Although there was no statistically significant difference in any of the demographic or injury characteristics between the intervention and control groups, there were significant differences in both the SCIM‐III and the SCIM‐SR‐Ar of the total sample. All participants completed the baseline assessment, rehabilitation course, and posttest after 6 weeks of assessment. They completed all the administered outcome measures. No adverse events were reported.

The intervention was administered by four licensed occupational therapists. Two were male, and two were female, with 8–20 years of clinical experience and 16 years of average experience in neurorehabilitation. Three occupational therapists held a bachelor′s degree, and one held a diploma in occupational therapy. All the participants received the planned minimum of 18 participation education sessions over 6 weeks. Total intervention time ranged from 352 to 435 min (M ± SD: 377.81 ± 25.44). Two‐thirds of occupational therapy sessions for PW‐SCI in the intervention group included participation education for 18.9 out of 60 min in place of adjunctive and active functional therapy, or 21.1% of allocated time for treatment.

For the analysis of adherence, the researchers received all 16 completed questionnaires, which were analyzed using descriptive methods. Commitment to the SADL‐eM was very high at 93.75%: None of the SADL‐eM content was deleted, added to, or changed. All participants in the intervention group used the SADL‐eM during each participation education session. None of the participants in the intervention group lost or spoiled the education manual. In contrast, 25.0% reported some inconvenience during intervention implementation due to the challenge in implementing the learned activities in the Gaza context, for example, environmental barriers. Additionally, 37.5% of the participants reported that there were elements of the SADL‐eM that do not apply to this patient category, such as driving rehabilitation and the implementation of the 1999 Disability Act, specifically the Right to Work Statement, accessibility, and assistive devices.

### 3.3. The Minimal Effect Size of the SADL‐eM

The SCIM‐SR‐Ar score for the total sample at baseline assessment ranged from 11% to 83% (M ± SD: 41.16 ± 19.82). The Kolmogorov–Smirnov test for normality showed normal data distribution (*t*: 0.867, *p*: 0.441). After 6 weeks of inpatient rehabilitation care, the SCIM‐SR‐Ar of the total sample improved by 16.06% (range: 6–93, M ± SD: 57.22 ± 22.80). This data was also normally distributed (*t*: 0.507, *p*: 0.959). Using a paired *t*‐test, there was a significant difference in the total means of SCIM‐SR‐Ar between baseline and after 6 weeks of inpatient rehabilitation (mean difference: 16.06, CI: 34.01–48.30, *t*: −5.77, *p* ≤ 0.001). Statistically significant improvement in all three domains of the SCIM‐SR‐Ar was also found (self‐care, respiration and sphincter management, and mobility) (*p* ≤ 0.001).

The total SCIM‐III scores at baseline assessment ranged from 11% to 93% (M ± SD: 39.63 ± 19.59). Using the Kolmogorov–Smirnov test, data were normally distributed (*t*: 0.867, *p*: 0.441). After 6 weeks of inpatient rehabilitation care, the SCIM‐III improved by 17.00% (range: 13–93, M ± SD: 56.63 ± 21.14). Data were also normally distributed (*t*: 0.507, *p*: 0.959). There was a statistically significant difference between baseline and posttest scores using the paired *t*‐test (mean difference: 17.00, CI: 32.56–46.69, *t*: −7.04, *p* ≤ 0.001). Similar to the SCIM‐SR, the subtotals of three domains of the SCIM‐III (self‐care, respiration and sphincter management, and mobility) also all improved significantly (*p* ≤ 0.001).

To compare the means of the SCIM after 6 weeks using linear regression analysis, first, the researchers used the independent group′s *t*‐test to adjust for the best measure at baseline. This was necessary because there was a significant difference between the means of the SCIM‐SR‐Ar (mean difference: 16.19, CI: 24.19–41.94, *t* = 2.50, *p* = 0.0182) and SCIM‐III (mean difference: 18.63, CI: 23.43–37.19, *t*: 7.04, *p* = 0.0051) in the intervention and control groups at baseline. A *t*‐test also showed a significant difference between the means of the SCIM‐SR‐Ar (mean difference: 19.94, CI: 35.39–59.11, *t* = 2.72, *p* = 0.0109) and SCIM‐III (20.38, CI: 36.98–55.89, *t* = 3.08, *p* = 0.0045) after 6 weeks, with the control group mean higher than the intervention mean. Linear regression analysis showed no significant difference between the intervention and control groups for either the SCIM‐SR‐Ar (CI: −4.82 to 19.93, *p* = 0.222) or SCIM‐III (CI: −4.91 to 17.31, *p* = 0.263) after 6 weeks of intervention.

Data of the Private and Organizational Religiousness subscales were collapsed into two categories for each question to perform suitable statistical tests, often/rare. The chi‐square test was used to adjust for the best measure at the baseline of the PRPS. There was a difference between the means of the intervention and the control group at baseline for the first question (How often do you pray: chi = 2.74, *p* = 0.098). There was no difference between the means of the other four questions at baseline. Using linear regression, the intervention had no significant effect on the PRPS items (*p* > 0.05).

Fisher′s exact test was used to adjust for the best measure at the baseline of the Organizational Religious Practices subscale. Using linear regression, the intervention had no significant effect on the Organizational Religious Practices subscale items (*p* > 0.05).

Data on driving and community mobility, health management, and religious rituals were collapsed into two categories for each question to perform suitable statistical tests: require assistance and independent. The chi‐square test was used to adjust for the best measure at baseline. There was no difference between the means of each item of driving and community mobility at baseline (6.681, *p* = 0.010). Linear regression analysis showed no difference between the intervention and control groups for driving and community mobility (*t* = −0.35, *p* = 0.729). There was no difference between the means at baseline for health management (participation in personal device care: chi = 3.16, *p* = 0.075; participation in health management and maintenance: chi = 2.24, *p* = 0.135) and religious ritual items (participation in Wudu: chi = 10.16, *p* = 0.001, regression: *t* = 1.05, *p* = 0.303; participation in Salat: chi = 10.16, *p* = 0.001, regression: *t* = 0.70, *p* = 0.492). The intervention used had no significant effect on the other ADL items (*p* > 0.05) (Table 3).

**Table 3:** Participation measures at baseline and after 6 weeks of intervention in the intervention and control groups (*n* = 32).

	Baseline mean (SD)		6 weeks mean (SD)		Between‐group difference mean (95% CI), *p*	Mean difference at baseline: Intervention and control	Effect size total population
	Intervention (*n* = 16) *m* *e* *a* *n* ± *S* *D*	Control (*n* = 16) *m* *e* *a* *n* ± *S* *D*	Intervention (*n* = 16) *m* *e* *a* *n* ± *S* *D*	Control (*n* = 16) *m* *e* *a* *n* ± *S* *D*			
SCIM‐SR‐Ar total (100)	33.06 ± 16.66	49.25 ± 19.87	47.25 ± 22.26	67.19 ± 19.15	Regression: CI: −4.82 to 19.93, *p* = 0.222	−16.19	16.06
Self‐care subtotal (20)	6.38 ± 4.94	9.94 ± 6.07	10.50 ± 6.64	15.63 ± 4.69	*t*‐test baseline adjustment: *t* = −1.82, *p* = 0.346	−3.56	4.90
Respiration and sphincter management subtotal (40)	20.19 ± 7.24	27.38 ± 8.37	24.56 ± 9.29	31.88 ± 7.68	*t*‐test baseline adjustment: *t* = −2.60, *p* = 0.123	−7.19	4.44
Mobility subtotal (40)	6.50 ± 6.70	12.63 ± 8.38	12.88 ± 8.80	19.69 ± 9.16	*t*‐test baseline adjustment: *t* = −2.28, *p* = 0.544	−6.13	6.72
SCIM‐III total (100)	30.31 ± 12.91	48.94 ± 21.01	46.44 ± 17.74	66.81 ± 19.68	Regression: CI: −4.91 to 17.31, *p* = 0.263	−18.63	17.00
Self‐care subtotal (20)	6.56 ± 4.99	10.94 ± 5.83	12.63 ± 6.24	16.00 ± 4.71	*t*‐test baseline adjustment: *t* = −2.28, *p* = 0.268	−4.38	5.56
Respiration and sphincter management subtotal (40)	20.25 ± 6.87	27.44 ± 9.43	23.75 ± 7.90	31.56 ± 8.10	*t*‐test baseline adjustment: *t* = −2.47, *p* = 0.187	−7.19	3.62
Mobility subtotal (40)	3.50 ± 4.20	11.94 ± 9.05	11.56 ± 7.19	19.81 ± 10.18	*t*‐test baseline adjustment: *t* = −3.38, *p* = 0.060	−8.44	7.97
Correlation at baseline: SCIM‐SR∗SCIM‐III							
Total					Pearson: 0.89		
Self‐care					Pearson: 0.82		
Respiration and sphincter management					Pearson: 0.81		
Mobility					Pearson: 0.85		
Private Religious Practices subscale					*p* > 0.05		
Organizational Religious Practices subscale					*p* > 0.05		
Driving and community mobility					*p* > 0.05		
Health management					*p* > 0.05		
Religious rituals					*p* > 0.05		
Private Religious Practices subscale							
How often do you pray?					Chi = 2.74, *p* = 0.098		
How often do you pray privately in places other than at church/mosque?					Chi = 6.00, *p* = 0.014 Regression: *t* = 0.35, *p* = 0.729		
How often do you watch or listen to religious programs on TV or the radio?					Chi = 5.58, *p* = 0.018 Regression: *t* = 0.00, *p* = 1.000		
How often do you read the Bible or other religious literature?					Chi = 4.07, *p* = 0.044 Regression: *t* = 0.00, *p* = 1.000		
How often are prayers or grace said before or after meals in your home?					Chi = 5.23, *p* = 0.022 Regression: *t* = 1.46, *p* = 0.154		
Organizational Religious Practices subscale							
How often do you go to mosque, church, or synagogue?					*p* = 0.250		
How often do you attend religious services: Regression?					*t* = 1.11, *p* = 0.275		
Besides religious services, how often do you take part in other activities at a place of worship?					*t* = 0.99, *p* = 0.331		

All the demographic and injury characteristics were homogeneous at baseline evaluation and insignificant (*p* > 0.05). However, it was noticed that the intervention group had more cervical injuries than the control group (five vs. four) and fewer lumbar injuries than the control group (six vs. seven). Also, the researchers noticed that the intervention group had more cases with complete SCI injury than the control group (two vs. one) and fewer cases with incomplete SCI than the control group (14 vs. 15). Dysfunction after SCI was less in the control group than the intervention group: paraplegia (two vs. six), paraparesis (10 vs. six), tetraparesis (three vs. four), and tetraplegia (zero vs. one). Better ASIA classification was observed in the control group than in the intervention group: ASIA A (two vs. five), ASIA B (one vs. two), and ASIA C (13 vs. nine).

## 4. Discussion

This pilot RCT aimed to determine if an RCT is feasible in this context and, if so, how the RCT design could be improved to maximize the likelihood of success. The researchers were also interested in the minimal effect of the SADL‐eM. The recruitment rate was satisfactory (70%); however, it was slow, with about 30 cases annually. The eligibility criteria were also implemented as predetermined in the study protocol [6, 7]. The randomization strategy was not appropriate. The retention rate was 100%, as all the participants who enrolled in the study completed all the pilot RCT phases. No inconvenience or difficulty was reported in the used outcome measures. Descriptive methods of analysis and statistical tests run were suitable for the study objectives and to test the research hypothesis.

The findings of this study highlight significant concerns about the feasibility of a full RCT as planned in our protocol [5]. Although recruitment, retention, application of eligibility criteria, and adherence to the treatment protocol met our predetermined criteria for feasibility, recruitment for the 32 participants took much longer than expected and was primarily from one hospital. The significant difference in SCIM scores between the intervention and control groups at baseline raises questions about the effectiveness of our randomization procedures, as the control group had less disability and dysfunction than the intervention group. Although the SCIM‐III and SCIM‐SR‐Ar worked well as outcome measures, they both measure limited domains of participation that do not fully capture the broad range of occupations that are important to PW‐SCI living in Gaza. The pilot study also confirmed the inappropriateness of our adapted versions of scales measuring religious participation.

The findings of this study suggest that the performance of participants in the daily activities improved over time in both intervention and control groups. Six weeks of inpatient rehabilitation care resulted in substantial improvement in the total score of the SCIM‐SR‐Ar of the total sample by 16.06% and the SCIM‐III total score by 17.00% [36]. Although there was a significant difference between the means after 6 weeks of intervention, the use of the SADL‐eM in occupational therapy did not explain this difference. There could be an improvement in other aspects, such as access to participation education resources, a change in the level of knowledge relevant to education participation of SCI, and participants′ attitudes to living with SCI. These aspects were not included in the outcomes of the pilot RCT, which rather focused on changing ADL skills necessary for survival and well‐being in the community. Another potential factor is that participants in the intervention group did not receive exactly the same input using the manual. Offering participants a choice in the section of the manual to focus on promoted person‐centered care, but this may have limited the consistency of the intervention for the purposes of an RCT.

The use of the SADL‐eM substantially increased the amount of participation education received by participants, and the adherence to the intervention among participants was very high, 63.5%–100%. Participation education for PW‐SCI in our intervention group was provided by qualified and experienced occupational therapists. It constituted 21.1% of allocated time for treatment with an obvious increase in time and frequency, up to three times compared to Foy et al. [38], who found that occupational therapists allocated 7.3% of their working hours for participation education of PW‐SCI. Participation education on SCI existed before the current intervention but was not well structured, as three of four readmitted participants had participation education during their previous admission.

The first problem the researchers encountered was the time taken to recruit the 32 participants over 13 months. Although the incidence of SCI in Gaza is 7/100,000 [39], it appears that many PW‐SCI in Gaza are not receiving any inpatient rehabilitation because of limited allocated budgets by the PMOH for rehabilitation (nearly 5% of the annual budget) and poor optional health insurance. The sample size calculation for the RCT indicated 90 participants are required to detect an effect of the SADL‐eM. In addition, 30 of the 32 cases were recruited from Hamad Rehabilitation Hospital. At the current recruitment rate, it will take an unacceptable 3 years to recruit the required participant sample. This prolonged timeline poses a critical threat by greatly increasing the risk of contamination, exposure, and maturation bias [40].

Clinical trials are not absolutely bias‐free; however, proper counteracting strategies such as blinding and randomization reduce possible sources of bias [40]. We attempted to counteract possible sources of popularity bias [40] while avoiding sample attrition, as SCI is a disease that has a low incidence [41]. Patients with SCI usually have a lengthy stay in inpatient rehabilitation settings and have greater opportunities to communicate with therapists, patients, and caregivers with potential maturation and contamination bias [40]. Moreover, both study groups were in the same department, which allowed the indirect opportunity for sharing information relevant to the study between patients and therapists. Using the SCIM‐SR‐Ar could be a source of exposure bias because it is a self‐rating outcome tool that indicates the best performance. Fortunately, and to our best knowledge, the SADL‐eM was the only participation educational intervention taking place in the study settings during our pilot RCT that limited the sources of exposure bias. The researchers also cooperated closely with an expert statistician to avoid statistical analysis bias.

It was difficult to use the religious subscales, as they were developed for Christian people; they do not have total scores; they are unsuitable in small samples, as it is difficult to perform meaningful statistical tests due to multiple categories; and they have a difficult interpretation of results [34, 35]. Also, Cronbach′s alpha was small (*a* = 0.43) for the PRPS in the feasibility study, which indicated very low reliability [5]. However, adding additional questions improved reliability to good in the pilot RCT (*a* = 0.74). There is a need to develop a culturally relevant religiousness outcome tool for inpatient rehabilitation that considers the gaps the researchers identified in the used tools.

Sensitive, holistic, and contextually relevant outcome measures are essential tools to evaluate and monitor SCI rehabilitation outcomes [42]. The SCIM‐SR and SCIM‐III are refined, validated, and worldwide implemented as primary functional recovery outcome measures for SCI [20]; however, they were criticized for lacking some domains of daily life activities, for example, driving and community mobility [5]. In the pilot RCT, the researchers used the SCIM‐SR‐Ar, which is culturally relevant and involved the voice of participants as an implementation of the participatory occupational justice framework [5]. However, future studies of the impact of interventions on participation for PW‐SCI should draw on the work of Nuuttila et al. [33] and Javanmard et al. [15] to select outcome measures that cover a wider range of participation domains.

The study findings indicate that the simple randomization method the researchers used failed to equally stratify the weight of injury characteristics between intervention and control groups because of the multiaspect nature of SCI. Thus, the randomization was biased in favor of the control group that had less dysfunction and disability characteristics and therefore better function. Block randomization is recommended in such studies where baseline scores may be affected by the level of disability and functionality, and especially in small samples [43]. The higher SCIM‐SR‐Ar and SCIM‐III total and subtotal scores of the control group at both baseline assessment and after 6 weeks of inpatient rehabilitation care may explain why the researchers found no evidence of the effectiveness of the SADL‐eM in comparison to usual care.

It is not surprising that some elements of the SADL‐eM do not apply to the participants (37.5%), for example, driving rehabilitation, the implementation of the 1999 Disability Act, and accessibility, since individuals were very early post‐injury and, in most cases, had not yet been involved in community occupations. However, participation education in some aspects of participation can be an entry to community inclusion after discharge.

According to the [44]) estimates and coordinated health assessments released in late 2025, there are over 2000 people living with SCI as a result of war‐related trauma in the Gaza Strip. Thus, this study addressed timely concerns of rehabilitation and occupational therapy care in times of disasters and wars. Nearly 50 countries in 2025 are torn by wars and armed conflicts, for example, the Gaza Strip, Syria, Lebanon, Ukraine, and Sudan. It is common now that parties of armed conflicts weaponize medication, food, and healthcare, including rehabilitation [45]. The 2 years of conflict (2023–2025) in Gaza were the most toxic, having erased the achievements and advancement of the last 56 years (1967–2023) and rendering it in massive need of rubble removal, deep reconstruction, and basic humanitarian survival needs. Preventable deaths in Gaza are increasingly the result of denied access to medical treatment. Healthocide describes the deliberate dismantling of health systems as a method of warfare against civilian populations. The targeting of healthcare in Gaza functions as a mechanism of social destruction that extends far beyond immediate physical harm [46]. The SADL‐eM can be a helpful tool to manage hundreds of people not only suffering from SCI sequela but also who are displaced and cannot find shelter, food, clean water, and rehabilitation care [47]. The SADL‐eM inspired new studies in Gaza using participation education by physiotherapists.

### 4.1. Limitations

There were five main limitations to this pilot RCT. The recruitment rate was slow and was subsequently expected to take a long time to conduct the main RCT, introducing more exposure, maturation, and contamination bias. Another potential threat of contamination bias is when adding an intervention to one group without the other, in terms of the effect of the intervention when compared to standard care. This bias pertains to the risk that the SADL‐eM might not be effective in future studies, as some subjects (study participants) may obtain and use the SADL‐eM as a personal initiative. Moreover, recruitment was mostly from one clinical setting, and the study took place in an inpatient rehabilitation setting, which provided an opportunity for participants and therapists to share information about ongoing interventions and thus introduced exposure and contamination bias. The sampling strategy was unsuitable and did not properly stratify case weight between the two study arms. Religiousness subscales were unsuitable for Muslims and difficult to run meaningful statistical analysis on, as the scales lack totals and the questions have many categories, up to nine choices. Other important aspects of participation education outcomes were absent, for example, access to participation education, level of knowledge, and participants′ attitude to living with SCI.

### 4.2. Implications for Research and Practice

This pilot RCT identified five significant barriers to our planned RCT. The slow recruitment rate was a barrier that raised the question of whether the RCT can be better planned as a community‐based intervention where there is a larger population for recruitment and where PW‐SCI have more opportunities to implement the information contained in the SADL‐eM at their own homes. The use of simple randomization was found to be inappropriate, and the researchers suggested blocking randomization to balance disability and dysfunction between the two study groups. There is also a clear need to develop a contextually relevant religious participation measure for people of the Islamic faith. The researchers found that the SCIM‐SR‐Ar could be an important tool to monitor and enhance occupational therapy care for PW‐SCI in a person‐centered way. Finally, future research needs to address absent aspects of participation education outcomes, that is, access to participation education, change in the level of knowledge, and participants′ attitudes to living with SCI.

## 5. Conclusions

Clinical trials are important to develop evidence‐based practices. Slow recruitment rates and an unsuitable sampling strategy were the main barriers to conducting a pilot RCT to evaluate a participation education intervention on participation in three clinical inpatient rehabilitation settings in the Gaza Strip. There were two rehabilitation settings, which rarely admit SCI for active rehabilitation, maybe a slow and silent healthocide. There was a change over time in all outcome measures used; however, this intervention did not have a statistically significant effect. These findings could be due to a small study sample. Given all the barriers in an inpatient clinical rehabilitation setting, the community may be a unique setting for such clinical trials. However, community‐based intervention would require the use of innovative technologies such as telerehabilitation or the development of a mobile app to deliver the intervention and collect data from participants. The SCIM‐SR‐Ar is working very well as a clinical outcome tool to be self‐administered, measure participation, and educate PW‐SCI about their potential in participation.

## 6. Key Findings

▪ Rehabilitation clinical settings are rich with data; however, conducting an RCT in such settings is difficult and bears many forms of bias.
▪ Rehabilitation services provided for PW‐SCI in the Gaza Strip have a substantial effect.

## 7. What the Study Has Added

▪ The community can be a unique setting for an RCT evaluating the effectiveness of participation education in SCI.
▪ The SCIM‐SR‐Ar and SCIM‐III are appropriate outcome tools to evaluate participation in PW‐SCI in the Gaza Strip. However, its use in the community is questionable, and many questionnaires or questions may return empty of answers.
▪ The study highlighted and addressed important, unique needs of Muslim PW‐SCI, that is, religious participation and relevant outcome tools.

## Author Contributions

The three authors (Moussa Kleib Abumostafa, Nicola Ann Plastow, and Maggi Savin‐Baden) equally contributed to all study aspects: initiating the study; developing the concept, design, methodology, protocol, and data analysis; and manuscript preparation. Moussa Kleib Abumostafa, Maggi Savin‐Baden, and Nicola Ann Plastow contributed equally to the study concept development, drafting and approving of the study protocol, designing methodology, statistical analysis, and drafting and approving the final version of the manuscript. Moussa Kleib Abumostafa was responsible for data collection.

## Funding

No funding was received for this manuscript

## Disclosure

The authors followed the appropriate guidelines for reporting health research and addressed all items recommended by the guidelines. The authors affirm that the manuscript is an honest, accurate, and transparent account of the study being reported; that no aspects of the study have been omitted; and that any discrepancies from the study as planned (and, if relevant, registered) have been explained.

## Ethics Statement

The study was approved by Stellenbosch University (HREC Project ID: 1635) and Helsinki Committee for Ethical Approval (PHRC/HC/689/20). This pilot randomized clinical trial uses an educational intervention that bears no risk for participants. FDA approval is not required.

## Consent

The authors ensured patients′ anonymity and that informed consent was obtained from each participant.

## Conflicts of Interest

The authors declare no conflicts of interest.

## Data Availability

The data that support the findings of this study are available on request from the corresponding author. The data are not publicly available due to privacy or ethical restrictions.
